# Integrating social science into oral health and dental research: a narrative review

**DOI:** 10.3389/froh.2025.1716748

**Published:** 2025-12-09

**Authors:** Frederick Howard, Nawal Jama, Duangporn Duangthip

**Affiliations:** Division of Dental Public Health, College of Dentistry, The Ohio State University, Columbus, OH, United States

**Keywords:** oral health, qualitative research, health disparities, interdisciplinary, social determinants of health, oral health fatalism, social science

## Abstract

Oral health outcomes are shaped not only by biological and clinical factors but by social and structural conditions. This narrative review synthesizes social science contributions to oral health research published between 2015 and 2025, situating them within a longer history of interdisciplinary engagement. Foundational social science concepts have informed contemporary frameworks of dental public health, yet their methodological and theoretical integration remains limited. Relevant studies were identified in *PubMed* and *AnthroSource*. Peer-reviewed, English-language articles that substantively engage oral health through qualitative approaches were selected for inclusion. Drawing on methods such as interviews, focus groups, and ethnography, this body of research highlights how cultural frameworks, institutional barriers, and lived experiences shape oral health practices and outcomes. Across this literature, three purposes for integrating social science emerge: (1) adding interpretive depth to quantitative data; (2) analyzing how behavioral logics interact with structural constraints; and (3) improving outcomes through culturally responsive interventions. In our conceptual synthesis of this work, we argue for the continued development of *bridge concepts* (e.g., oral health fatalism, tooth shame, and status passage), which enable collaboration across disciplines by linking clinical practice to social context. Social science approaches expand explanatory frameworks, deepen contextual understanding, and illuminate oral health as a social and ethical phenomenon. By moving beyond methodological borrowing toward co-production of knowledge, interdisciplinary collaboration can more effectively address oral health inequities and support the development of care that is both scientifically rigorous and socially responsive.

## 1 Introduction

Oral diseases are often investigated through biological and clinical frameworks, yet their most persistent causes are social in kind. Oral health outcomes are shaped by deeply embedded social, economic, and civic institutions. Increasingly, dental researchers and public health professionals recognize the value of social science theories and methodologies in addressing oral health disparities. Institutions such as the World Health Organization and the U.S. Department of Health and Human Services have emphasized the role of social determinants in the development of dental caries and periodontal disease—two of the most prevalent chronic conditions globally (1–3).

Despite growing recognition of the importance of social determinants in oral health, the integration of social science perspectives into dental research remains inconsistent and fragmented. Prior reviews have advanced our understanding of interdisciplinary collaboration between social science and oral health research, but a broader synthesis is still needed to situate these contributions in their historical and contemporary context (4–6).

Recent initiatives such as the 2020 Behavioral and Social Oral Health Sciences Summit and the *2022 Consensus Statement on Future Directions for the Behavioral and Social Sciences in Oral Health* expertly highlight the value of cross-disciplinary collaboration (7, 8). Yet there remains an opportunity to investigate the development and integration of methods over the last decade. Critical gaps remain in understanding how to effectively bridge social science theory and dental and oral health research practice, and to identify productive tensions that both challenge and enrich interdisciplinary collaboration.

This narrative review addresses these gaps by presenting a wide-ranging analysis of the intersection between social science and oral health research. It aims to benefit researchers, educators, and policymakers seeking to advance comprehensive, socially informed oral health research and underscores the potential of integrated approaches to improve population-level oral health outcomes.

Specifically, this paper aims to: (1) provide an historical overview of social science engagement with oral health, tracing key milestones and intellectual shifts; (2) highlight contemporary commentary on interdisciplinary research; and (3) synthesize recent studies published between 2015 and 2025 by population type, identifying key research topics, common methodologies, and productive tensions. We also argue for the development of *bridge concepts* to facilitate collaboration across disciplinary boundaries.

Oral practices are locally situated and often shared. Examining the cultural contexts in which our teeth are viewed, judged, diagnosed, cleaned, and cared for is an integral part to any inquiry aiming to improve outcomes. Examining the diverse ways people attribute meaning and significance to their teeth is critical for a deeper understanding of oral health trends. This review treats culture not as a fixed attribute or simple demographic variable, but as a dynamic set of meanings and practices through which people interpret the mouth, teeth, the body, and the significance of care. Cultural perspectives shape how pain is expressed, how prevention is valued, and how clinical encounters are experienced. Recognizing these processes is essential for understanding oral health as both a biological and a social phenomenon—and for integrating behavioral, clinical, and policy perspectives within a common analytic frame.

## 2 Historical background and established intersections

Social science has long shaped the conceptual foundations of public health, including dentistry. In the 19th century, well before the emergence and adoption of the concept of *social determinants of health* (SDoH), Virchow and Durkheim described how social structures shape health outcomes through labor, inequality, and education (9, 10). Weber's formulation of *socioeconomic status* (SES) linked flourishing to access to power and resources instead of income alone (11, 12). Later, Canguilhem reframed health as an adaptive capacity rather than a fixed biological state (13), and Foucault analyzed how institutions and discourses govern health (14–16). Bourdieu's concepts of *habitus* and *capital* further explained the patterned inequalities in care-seeking and access (17, 18). Although rarely cited directly in dental research, these perspectives underlie familiar terms such as *social capital*, *health equity*, and *social determinants*.

Dentistry has engaged social science in its own way: Cohen and Richards' systematic review of social science in dentistry (1955–1970) emerged in the context of debates on water fluoridation, focusing on how institutional structures shaped public acceptance (19, 20). The 1971 *Milbank Memorial Fund Quarterly* special issue, “Toward a Sociology of Dentistry,” examined dentistry as a professional institution, analyzing delivery systems, payment models, gendered labor, and relationships with insurers and government agencies (21–24). By the late twentieth century, medical sociologists and public health scholars applied these insights to health disparities, culminating in the WHO Commission on Social Determinants of Health, which defined SDoH as “the conditions in which people are born, grow, live, work and age.” (2, 25).

Since then, the focus has shifted from dentistry as an institution to the lived experience of patients. The 2016 FDI World Dental Federation's redefinition of oral health explicitly incorporated psychosocial dimensions as well, and the 2020 Behavioral and Social Oral Health Sciences Summit signaled renewed commitment to interdisciplinary, qualitative engagement (7, 26). These developments underscore that the social sciences have long theorized health, but their meaningful methodological integration into dental research remains comparatively fragmented.

## 3 Expansion of social science themes and approaches in dentistry

The Summit resulted in a special issue of the journal *Community Dentistry and Oral Epidemiology* in which McNeil and Randall identify two persistent barriers to the development of social science within oral health research (7). First, they note the absence of sustained, long format conferences that allow interdisciplinary dialogue to unfold beyond brief or fragmented exchanges. Second, they highlight the lack of international platforms capable of amplifying these conversations across diverse contexts. Building on their observations, we suggest that deeper epistemological tensions—particularly between biomedical and sociological paradigms—have also complicated the field's trajectory.

Across prior publications in the field, social science is often paired with or conflated with behavioral science. This recurring association warrants reflection. In oral health research, behavioral science—typically rooted in psychology—has emphasized individual-level interventions and frameworks such as the *Health Belief Model* and *Social Cognitive Theory* (27–29). Social science, by contrast, as developed most cogently in sociology and anthropology, tends to situate oral health within broader frameworks of meaning, social structure, and context. While the two orientations sometimes overlap, they generally operate at different levels of analysis and pursue different explanatory aims. In practical terms, this means that behavioral approaches tend to design interventions that modify individual knowledge, attitudes, or habits, whereas social science approaches more often target the conditions that shape those behaviors in the first place.

The Summit's resulting special issue reflects this dual emphasis. It promotes rigorous behavioral strategies to improve patient outcomes, while also engaging broader questions of inequality and social constraint in oral health (30–32). Increasingly, qualitative and mixed methods are valued for their ability to illuminate the “different worlds in which people live and work” (33), supporting more culturally responsive interventions (34, 35).

The *2022 Consensus Statement on Future Directions for the Behavioral and Social Sciences in Oral Health* advances a scalar framework for interdisciplinary research. As McNeil et al. write, “A comprehensive understanding of [oral health promotion, service delivery, and equity] requires consideration of influences at individual, family, community, group, regional, national, and global levels.” (8) This scalar approach foregrounds the multiple sites of intervention that oral health research must address.

*The Consensus Statement* outlines four key directions. First, it calls for more *middle-range* approaches that connect proximal behavioral causes with distal structural determinants—bridging the gap between individual action and systemic constraint. Second, it advocates for methodological pluralism, emphasizing that methods should be scaled to the research question and context. Third, it encourages exploration of underexamined social environments as potential sites for intervention, e.g., public housing and digital platforms. Finally, it promotes robust dissemination strategies that prioritize stakeholder engagement, contextual sensitivity, and methodological precision.

These directions are strategic and promising, but they also raise critical questions. Why do so many interdisciplinary ideals remain unrealized? What barriers inhibit collaboration? How should social scientists respond to clinicians who remain skeptical of social research? And how are methodological disagreements negotiated within interdisciplinary teams?

Jones and Gibson's 2022 edited volume *Cultures of Oral Health* showcases the breadth of contemporary social science inquiry in oral health (36). Topics include professional ethics, cosmetic dentistry, gendered career pathways, historical politics of advertising, and cultural representations of teeth. These studies highlight how oral health is shaped by symbolic meaning, embodiment, and power.

Interdisciplinary collaboration is increasingly central to oral health innovation. Community-based partnerships demonstrate how local knowledge and public health frameworks can improve equity. These collaborations also reveal persistent challenges: disciplinary hierarchies, divergent epistemologies, and entrenched institutional practices.

In the next section, we explore contemporary qualitative investigations in oral health published over the last decade, with particular attention to cultural frameworks and the productive frictions that arise in interdisciplinary collaboration.

## 4 Interdisciplinary frictions and cultural frameworks in oral health (2015–2025)

A substantial body of oral health research published between 2015 and 2025 is dedicated to understanding how cultural beliefs and practices shape oral health outcomes. These investigations vary in scope and focus, but all are underpinned by a recognition that social norms are deeply embedded aspects of human life. These norms not only influence the experience of oral disease but also affect prevention, access, and treatment. This section examines recent contributions of qualitative methods to oral health research, considers the frameworks used to describe relevant social relations, and builds on earlier scholarly calls for interdisciplinary collaboration (4–6, 37). The review emphasizes how qualitative and interpretive approaches—particularly interviews, focus groups, and ethnography—illuminate lived experiences of oral health and the conditions that shape them.

### 4.1 Search strategy and inclusion criteria

To ensure comprehensive coverage of social science research in oral health, we conducted a structured search of *PubMed* and *AnthroSource* on July 14, 2025. The search employed combinations of the following terms and their derivatives: “oral health,” “dentistry,” “teeth,” “mouth,” “sociology,” “anthropology,” “ethnography,” “interview,” “participant observation,” and “focus group.” Boolean operators were used to combine terms across disciplinary and methodological domains [e.g., oral health AND (ethnography OR anthropology OR sociology)]. When applicable, both *MeSH Terms* and *All Fields* search specifiers were used.

From this initial search, a set of inclusion criteria was applied. Eligible papers were peer-reviewed, full-text English-language publications presenting original human-subject research that substantively engaged oral health through social science methodologies, and were published between January 1, 2015, and June 30, 2025. Exclusion criteria omitted studies focused solely on forensic or archaeological contexts. Papers that used demographic descriptors of ethnicity or race without engaging shared meaning or experience were also excluded. Retrospective analyses or studies that collected data exclusively through surveys, questionnaires, telephone interviews, focus groups with fewer than 20 participants, or interviews with fewer than seven participants were excluded, as were other reviews (e.g., systematic, scoping, or narrative). Finally, papers employing qualitative methods only to assess the validity of a research instrument or framework were omitted.

The search initially yielded 4,123 articles. After removing duplicates, applying inclusion and exclusion criteria, and conducting full-text review, 65 papers were included in the final synthesis. Consistent with standards for narrative reviews, discussed papers were appraised for their methodological approach, conceptual relevance, and regional scope, rather than formal quality scoring.

A note on the criterion of qualitative methods: this review is specifically concerned with the application of social science methodologies in oral health research. Many papers describe their use of *qualitative* methods without demonstrating meaningful engagement with social science theory or practice. During the search process, it became evident that the term encompassed a range of approaches—from structured questionnaires and brief spoken surveys to in-depth ethnography. Throughout this review, *qualitative* is used in the stricter sense of social science investigation, characterized by semi-structured interviews, ethnographic observation and participation in the social context under study, and focus groups with adequate participant depth. Several short interview or small focus group studies were omitted, reflecting both the field's growing interest in participants' perspectives and the need to strengthen the application of qualitative methods in oral health research.

### 4.2 Key research topics using social science methods in oral health research

Over the last decade, clear patterns have emerged in the topics, populations, and analytic orientations of qualitative oral health studies. From these patterns, five key research topics were identified that capture the major directions of current social science inquiry in oral health: (1) parental perspectives and maternal care; (2) immigrant and refugee oral health; (3) elder care and aging; (4) social analysis and institutional critique; and (5) cultural practices as assets for intervention. While some articles in these categories overlap conceptually, together they chart the primary terrains in which social science perspectives are shaping oral health research today—from intimate domains of caregiving to institutional structures and public health practice. Notably, the majority of studies were conducted in high-income, Western contexts, with emerging but still limited representation from low-income populations in Asia, Africa, and South America.

After reviewing the literature, individual interviews and focus groups were the most used qualitative methods. They are particularly well suited to investigate both known and under-examined cultural and behavioral factors within the established disciplinary time constraints. Ethnography is also represented in the reviewed articles with increasing prominence. Perhaps because of current funding structures and disciplinary conventions, investigations in oral health research tend to be shorter and more sporadic than ideal ethnographic conditions demand. Mixed methods studies are less common, but are increasingly employed to triangulate findings and enhance depth of understanding (34). Ongoing efforts to create co-produced, mixed methods designs that integrate diverse epistemologies across study design, analysis, and dissemination are essential for improving the interpretive depth and translational impact of oral health research (33, 38).

#### 4.2.1 Parental perspectives and maternal care

Childhood oral health remains the central focus of qualitative oral health research, representing nearly half of the unique returns of the search. Because parents and caregivers are the gatekeepers of consent, they are integral to understanding the development of children's behaviors and affective orientations. Aside from the few studies that interviewed adolescents directly (39, 40), parents are the primary interlocutors for investigating childhood oral health.

The most common aim of this category is the investigation of *parental perspectives* on child oral health more broadly (41–45). McLean et al. describe “natural parenting” movements that promote sugar free diets, yet eschew fluoride, positioning oral health within broader identity projects that resist biomedical authority (46). A comprehensive, multi-sited study by Burgette et al. traces the circulation of fluoride misinformation among mothers making decisions about their children's oral health, illustrating how trust and expertise are negotiated within contemporary health information networks (47). Using a combination of interviews, participant observations, and a focus group, Balasooriyan et al. highlight how norms of Dutch families intersect with economic disadvantage to shape children's oral health trajectories (48). Articles within this category demonstrate that parental decision-making is not only a matter of individual preference but is embedded in cultural logics, structural pressures, and social identity that directly shape outcomes.

A variant of the *parental perspectives* theme describes the beliefs and behaviors of ethnic minority and Indigenous parents (44, 49–51). Davis et al. compared white and Hispanic parents seeking orthodontic treatment for their children, showing how decisions were shaped by intersecting values, expectations, and aspirations (52). Walker et al. investigate Midwest American Latino caregiver's perspectives on the inevitability of childhood oral disease and gendered expectation of responsibility for oral care (53). The focus of this subset is not migration but the recognition of culturally specific practices and beliefs that operate among dominant social norms.

Challenges experienced by parents of children with disabilities or neurodivergence are represented in recent literature. Alwadi et al. take an ethnographic approach to studying children with disabilities in Saudi Arabia (54), while Junnarkar et al. and Cai et al. examine the perspectives of parents of children with autism (55, 56). These studies are not focused exclusively on disability; they detail parents' perspectives on their children's challenges, highlighting how caregiving intersects with structural barriers to care.

Studies on parental perspectives examine caregiving practices as well as the cultural logics that connect childbearing to oral health outcomes such as tooth integrity (57). Some papers focus specifically on how parents perceive dental providers and oral health promotion programs (58–61). Cruz et al. identify factors that build and erode trust between patients and dentists, showing that the dentist–patient relationship is shaped by experiences of choice, control, and support (62). Expertise is not an immutable attribute of providers but something negotiated through interpersonal dynamics and prior encounters with care.

#### 4.2.2 Immigrant and refugee oral health

Research on immigrant and refugee populations highlights how oral health is mediated by migration histories, cultural expectations, and systemic barriers. Nearly all the studies on this topic involve parents reflecting on their children's oral health, with an important distinction from the perspectives of ethnic minority parents discussed above. These studies position parents as entry points through which divergent cultural beliefs and practices encounter broader community norms, while their children often represent processes of alignment through acculturation. Central to this research topic is the suggestion that cultural features can be barriers within oral health care systems. The family emerges as a key social structure mediating between individuals and institutions, particularly in migrant and economically under-resourced communities where structural and cultural pressures converge (63–66).

Studies highlight how language barriers and bureaucratic complexity hinder access to care for non-English-speaking mothers living in Anglophone countries (67, 68). Adebayo et al. explore the perspectives of African migrant caregivers in an Australian elderly care facility (69). These studies reveal how processes of cultural adjustment shape the experiences of both patients and care givers. They demonstrate the need for culturally responsive care and establish how oral health is entangled with negotiations of belonging, expertise, and access within shifting social expectations and institutional constraints. Calvasina et al. persuasively argue that improvements in material conditions—such as access to affordable care—often exert greater influence on outcomes than deficits in acculturation or cultural assimilation (70).

#### 4.2.3 Elder care and aging

Among older adults, oral health research has increasingly foregrounded affective and embodied experiences, knowledge, and beliefs (71, 72). Gerontological studies often draw on phenomenological frameworks for interpretation and show a growing interest in ethnographic methods for data collection. Folker et al. introduce the concept of *tooth shame* in Danish elder care, showing how aesthetics and emotion influence both the willingness to seek treatment and the quality of interactions with providers (73). Gibson et al. conceptualize full edentulism as a *status passage*—a transition into new social identities tied to aging or marginalization (74). Folker also explores *dental disgust*, identifying how care is diminished in institutional settings when staff members' aversion to oral decay interferes with treatment (75). These works argue for oral health frameworks that account for function as well as dignity, affect, and social participation.

In gerontology, qualitative inquiry has long illuminated subjective experience and contextual meaning. Cross-disciplinary collaboration in this field offers particularly fruitful models of inquiry, demonstrating that aging is understood not merely as decline but as a social process and lifelong experience (76, 77). MacEntee et al. examine the life practices of self-regulation and self-care among elderly Chinese participants in Hong Kong and Guangzhou, exploring factors like traditional medicine, family, and the effects of the commercialization of dental services (78). Other studies highlight broader barriers to care experienced by under-resourced elderly populations (79–82). This key research topic underscores how oral health in later life is not only a matter of clinical intervention but a reflection of social value, institutional capacity, and the moral economies of care.

#### 4.2.4 Social analysis and institutional critique

Social science research does more than map cultural practices—it also critiques the tendency to over-attribute poor outcomes to cultural difference when structural inequalities are often more determinative. This key research topic foregrounds class and economic precarity as upstream determinants of oral health and interrogates how the field of dentistry itself participates in reproducing social hierarchies. Khalid and Quiñonez critique cosmetic dentistry, showing how whitening and orthodontics reinforce racialized and class-based norms of beauty, deepening stigma for those who cannot or choose not to conform (83). Similarly, Wickström's ethnographic account, “I Hope I Get Movie-Star Teeth,” highlights orthodontics as a site of aspiration where ideals of normalcy remain out of reach for most patients (84). These studies call attention to the risks of cultural essentialism in oral health research. By framing structural exclusion as “cultural difference,” clinical discourse can inadvertently obscure the inequities it seeks to resolve.

Research in correctional settings reveals how the organization of institutional life shapes oral healthcare delivery (85). The studies describe fragmented care, limited continuity, and logistical barriers, but also identify opportunities for reform through person-centered and rights-based approaches. Similar structural themes emerge in research on homelessness, where Durey et al. show that community-centered models can improve engagement and reduce stigma yet are often undermined by bureaucratic inflexibility and inconsistent funding (86). Indigenous oral health research situates access within historical and policy contexts, highlighting how colonial histories, mistrust of institutions, and structural neglect continue to shape oral health inequities (87, 88).

Complementary policy-oriented studies further situate oral health disparities within systems of governance and resource allocation. Etiaba et al. analyze the political and institutional processes shaping oral health policy development in Nigeria, illuminating how power dynamics among actors and institutions constrain reform (89). Mohammadpour et al. likewise find that resource shortages and disjointed policy, rather than community behavior, constitute the primary barriers to care in Iran (90). These studies demonstrate that meaningful progress in oral health equity depends as much on confronting institutional and political arrangements as on modifying individual practices or beliefs.

#### 4.2.5 Cultural practices as assets for intervention

Another line of inquiry explores oral health practices that emerge outside formal institutions of care. Rather than viewing culture as a barrier to biomedical care, recent qualitative research demonstrates how preexisting practices and knowledge systems can serve as resources for improving oral health outcomes (91, 92). Shrivastava et al. advance the concept of *two-eyed seeing* to describe the divergent priorities of Cree and non-Cree providers, integrating Indigenous knowledge systems with biomedical approaches in Cree communities (93). Rather than treating Indigenous practices as barriers, a growing body of scholarship emphasizes them as assets that sustain oral health through meaningful frameworks of well-being (51, 88, 94–97).

These approaches offer epistemological alternatives to dominant biomedical models, expanding the kinds of questions researchers can ask about motivation, trust, and cultural influence. Using qualitative methods, Laird et al. trace the social life of the Somali miswak, demonstrating the ways oral hygiene tools are situated within cultural meaning and transnational histories, and how existing traditions of oral care can inform our understanding of oral health (98). Concepts like *two-eyed seeing* create opportunities for collaboration across knowledge systems. They encourage clinicians to view Indigenous practices not as competing explanations but as parallel logics of health that can align with preventive and restorative goals. In doing so, they illustrate how co-production of knowledge becomes possible when multiple traditions of care are recognized as legitimate contributors to oral health.

### 4.3 Outliers and the limits of the narrative review

Not all social science oral health research fits neatly into the thematic categories outlined above. Some studies engage experiences or behaviors that cut across multiple areas of inquiry making them difficult to situate within a single topic. Patterson-Norrie et al. document the perspectives of patients with eating disorders, revealing how oral health concerns intertwine with complex bodily and affective experiences (99). Musa et al. investigate the integration of dental services into health and social care systems for people with complex needs—a category that spans multiple vulnerabilities (100). Studies focused on disabilities extend this analysis to the embodied challenges of care, describing how barriers to access are compounded by communication difficulties, sensory sensitivities, and provider discomfort (101, 102). These studies highlight the breadth of social science engagement with oral health, extending beyond the key research topics we have presented.

Despite the utility of narrative reviews for describing complex subjects in conceptually rich ways, they remain limited by potential author bias, lack of exact replicability, and the possibility of selective or incomplete article inclusion. While our search was rigorous, the innovation of this review lies in its conceptual synthesis rather than data collection. Although most qualitative oral health studies originate from high-income or Western contexts, this review highlights scholarship from non-Western contexts to improve global representation. This uneven distribution of research reflects the field itself and underscores the need for sustained investment in qualitative and interdisciplinary oral health research in underrepresented regions, where sociocultural and structural determinants remain comparatively undocumented.

### 4.4 Bridge concepts as tools for co-production

Some qualitative research efforts in public health aim to map the social terrain for intervention; others are more critical, describing how dental institutions themselves reproduce norms that sideline already vulnerable groups. This divergence in approach reflects broader tensions in the interdisciplinary field. Some researchers use cultural insight to improve behavioral interventions, while others treat culture not as a variable to manage but as a source of established, meaningful practice and knowledge.

**Figure 1:**
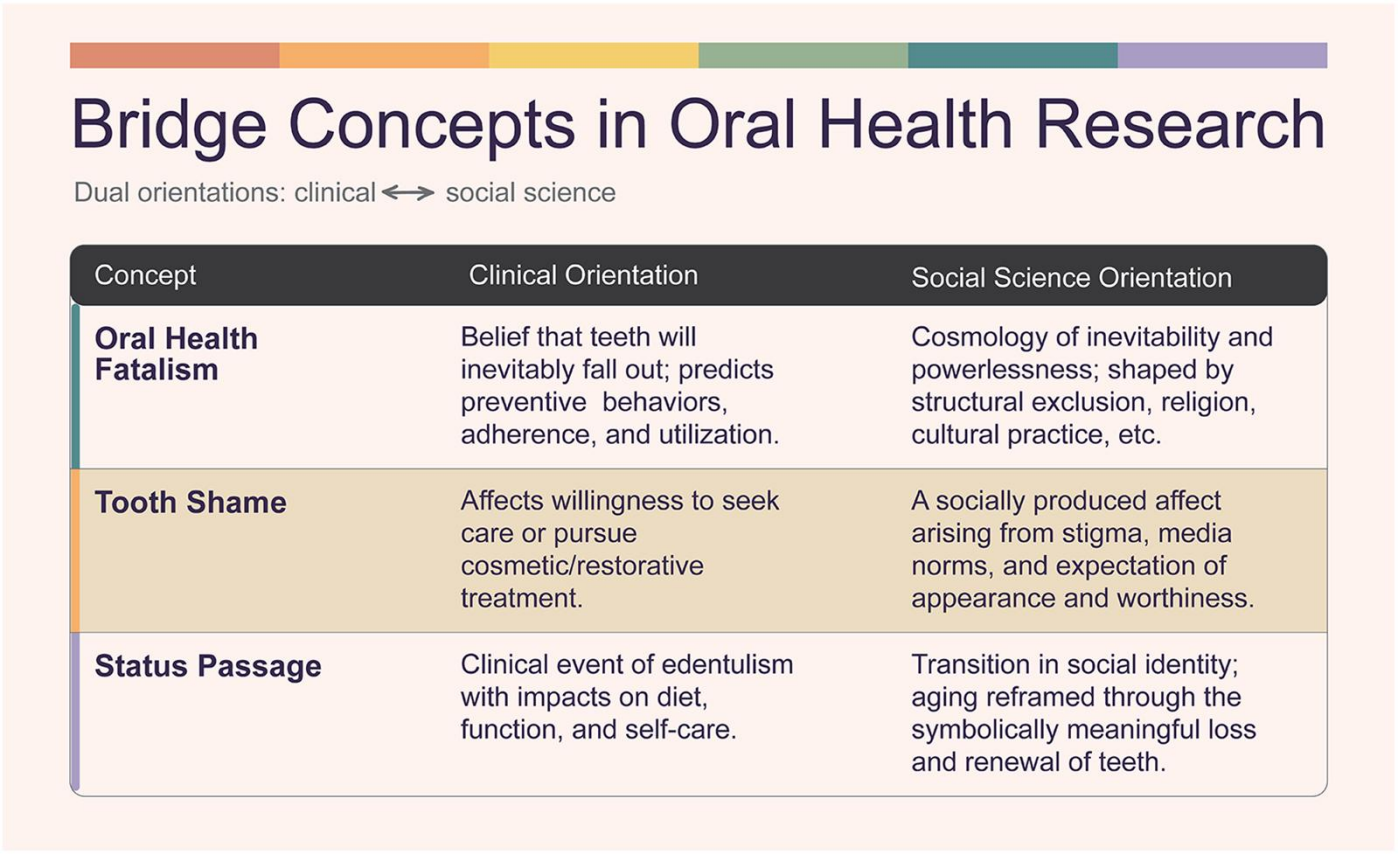
Conceptual orientations beneficial to clinical and social science research.

To aid interdisciplinary collaboration, we propose the continued development of *bridge concepts* as exemplified by *oral health fatalism* and *tooth shame*. These terms do more than describe behavior—they interpret it within cultural, affective, and structural contexts. Figure 1 maps the conceptual orientations linking clinical, behavioral, and social-scientific approaches, illustrating how bridge concepts mediate between individual behaviors and structural determinants. Bridge concepts enable collaboration by holding disciplinary logics together within the same analytic frame. For example, *oral health fatalism* has been studied by Hammersmith et al. in relation to caregivers of children with autism spectrum disorder and in broader pediatric populations, exploring how beliefs about inevitability and control shape oral health practices (103, 104). Though Hammersmith et al. do not incorporate primary qualitative work in their investigations into fatalism, their conceptual framing strongly invites social science methodologies and analysis. Hoffman et al. demonstrate how fatalism intersects with race, class, and access in low-income African American communities (105). Bridge concepts offer shared vocabularies for researchers across fields and help illuminate the lived experience of oral health.

For the clinician, *oral health fatalism* is an empirical variable: does the belief that teeth inevitably fall out reduce preventive behaviors or discourage treatment adherence? For the social scientist, fatalism signals a broader cosmology of inevitability and powerlessness, often rooted in structural exclusion, religious worldviews, or cultural practice. Both orientations are valid but ask different questions of the same phenomenon. As a bridge concept, fatalism allows alignment: clinicians situate patient beliefs within cultural practice, while social scientists connect meaning and power to health outcomes.

Other examples include *tooth shame* (73)—clinically relevant for its effect on utilization, and socially significant as a stigmatized affect shaped by cultural norms—and *status passage* (74), where dental status is both a clinical event and a socially meaningful transition in identity. Bridge concepts avoid reduction to either variables in regression models or purely cultural constructs. Instead, they establish common ground for interdisciplinary collaboration, moving research beyond methodological borrowing toward co-production of knowledge.

While these collaborations underscore the value of social context in care delivery, they also expose the persistent challenges of interdisciplinary work. Differences in epistemology, communication style, and disciplinary hierarchy can inhibit the integration of social science into clinical education and research. Yet these tensions are not merely obstacles—they are productive. As anthropologist Anna Tsing defines frictions, “the awkward, unequal, unstable, and creative qualities of interconnection across difference,” they generate opportunities for reflexivity, institutional critique, and more expansive forms of inquiry (106).

The structural position of dentistry itself complicates these collaborations. Dentistry's longstanding separation from medicine and its partial alignment with the personal service sector have shaped how oral health is perceived, accessed, and funded (107). As Elizabeth Mertz argues, the enduring “dental–medical divide” presents challenges for researchers and clinicians alike, especially in a moment of technological transformation (e.g., telehealth, machine learning) and growing pressure for interprofessional cooperation (108).

Bridge concepts offer analytical traction but require careful operationalization to avoid the pitfalls of terms like CaLD (Culturally and Linguistically Diverse), which are often used uncritically to vaguely index non-Western, non-European or Anglo origins, obscuring structural inequalities. Scholars such as Calvasina et al. rightly critique the overuse of cultural labels without sufficient theoretical grounding (70). In practice, such concepts can be operationalized by designing studies that integrate qualitative accounts of experience with quantitative or biomedical indicators—for example, pairing interview-based narratives of stigma or care-seeking with clinical measures of oral health outcomes. This approach does not attempt to quantify culture or meaning; rather, it situates these dimensions as practical components of evidence, allowing multiple forms of knowledge to speak to one another. As part of a translational research agenda, bridge concepts function as intermediaries between social inquiry and clinical application, guiding the design of interventions and the interpretation of outcomes in ways that remain sensitive to lived experience.

The health sciences too have developed frameworks that recognize the interrelated variables that govern health outcomes. *Complexity theory* is a highly relevant approach that accounts for the dynamic interplay of biological, behavioral, and social processes (109). For oral health research, this orientation underscores that individual outcomes are shaped simultaneously by biological factors, structural inequalities, clinical systems, and cultural norms. Key to this insight is that there are multiple feedback loops that inform the outcomes of complex systems.

Bridge concepts help clarify how social, material, and biological processes interact dynamically over time. They encourage researchers to conceptualize oral health as an emergent property of interconnected relationships—linking individual behavior, social context, and policy environment—rather than as a set of isolated clinical events.

Across the reviewed studies, theoretical frameworks were used unevenly and often implicitly. Future research would benefit from making these theoretical orientations more explicit. While qualitative techniques can be applied productively across disciplines, it is important to recognize that social science methodologies are shaped by the theories from which they emerge. Attending to this connection can deepen interdisciplinary collaboration, reflect a shared investment in the intellectual foundations of the methodology, and enhance the interpretive power of qualitative inquiry in oral health research.

As oral health research continues to evolve, interdisciplinary collaboration ought to improve the discrete toolkit model and move toward co-production. Social science is not merely a supplement to clinical inquiry—it has the potential to reshape how oral health is understood, taught, and practiced. This will require institutional commitment, curricular reform, and a willingness to engage with complex relationships that eludes clear generalization.

**Figure 2:**
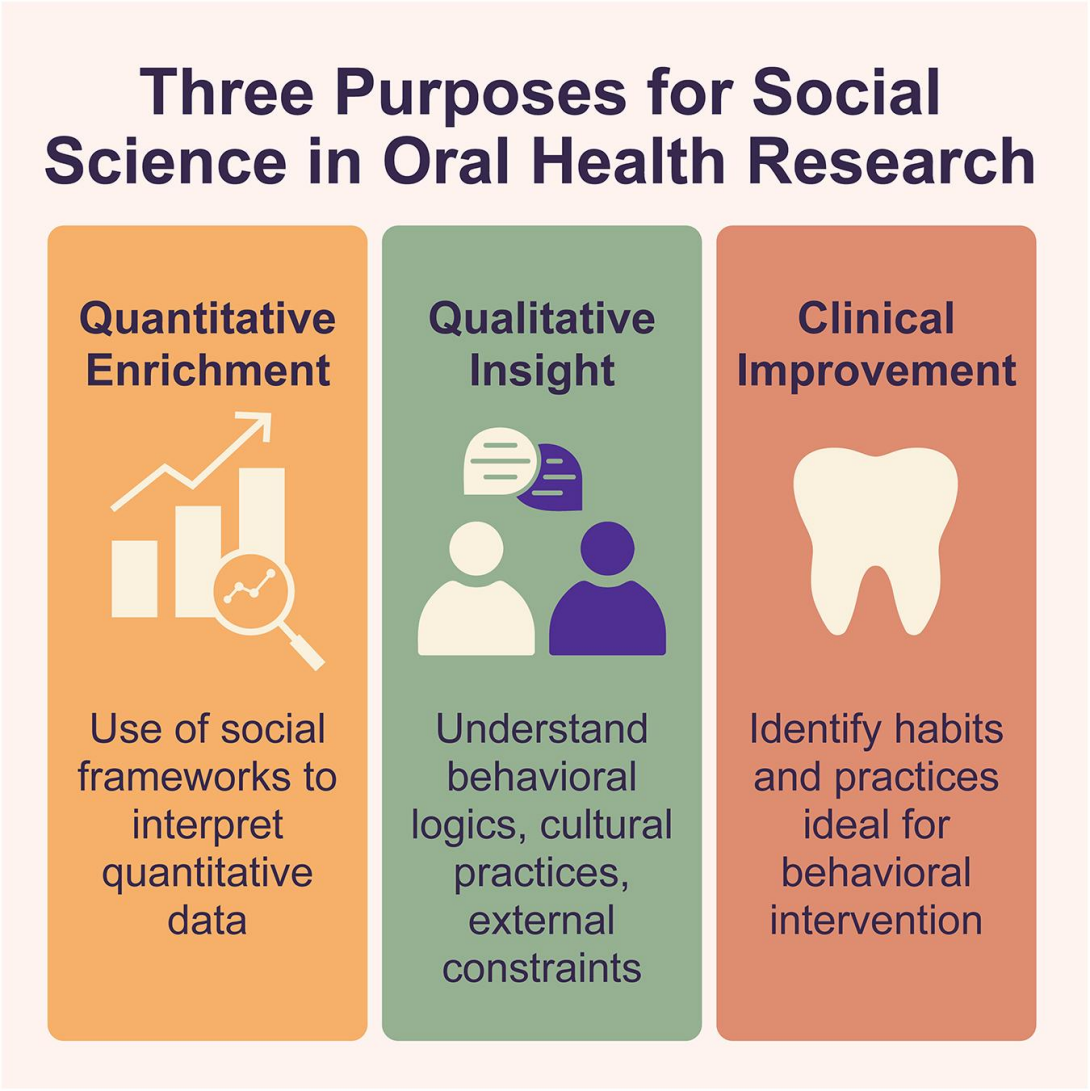
Three modes of oral health research using social science methodologies.

Based on our review of articles published from 2015 to 2025, three primary purposes for integrating social science into oral health research emerge resulting in distinct types of research (Figure 2). First, some researchers use social frameworks to enrich quantitative data and add interpretive depth. Second, social science methods are valued for their ability to understand behavioral logics, cultural practices, and institutional constraints that shape oral health outcomes. Third, social and behavioral sciences are often mobilized to improve clinical outcomes, particularly through the identification of behaviors and practices most ideal for intervention. Figure 2 depicts these three modes, illustrating the spectrum of interdisciplinary collaboration, from studies that apply social theory conceptually to those that embed social methods within clinical and public-health designs. Similar functions of qualitative research have also been outlined elsewhere (110).

These motivations reflect distinct epistemological commitments as to what counts as evidence. Clinicians often seek to identify levers for behavioral change—reducing sugar intake, increasing fluoride use, or improving hygiene practices. Social scientists, by contrast, situate behavior within broader structures of constraint and meaning. At stake is the *locus of control*: are the most actionable variables for improved outcomes managed by individuals or the networks and environments that shape their choices? In the literature, locus of control is posed as an internal-external binary, where *internal locus of control* refers to a person's belief that they have the capacity to positively affect their health outcomes, whereas *external locus of control* refers to a person's belief that their outcomes are a result of chance, fate, or powerful others (104, 111, 112). Typically, a defined locus of control is a commentary on the belief of the patient, but it is also undoubtedly a construct advanced by researchers to describe sites of intervention.

**Figure 3:**
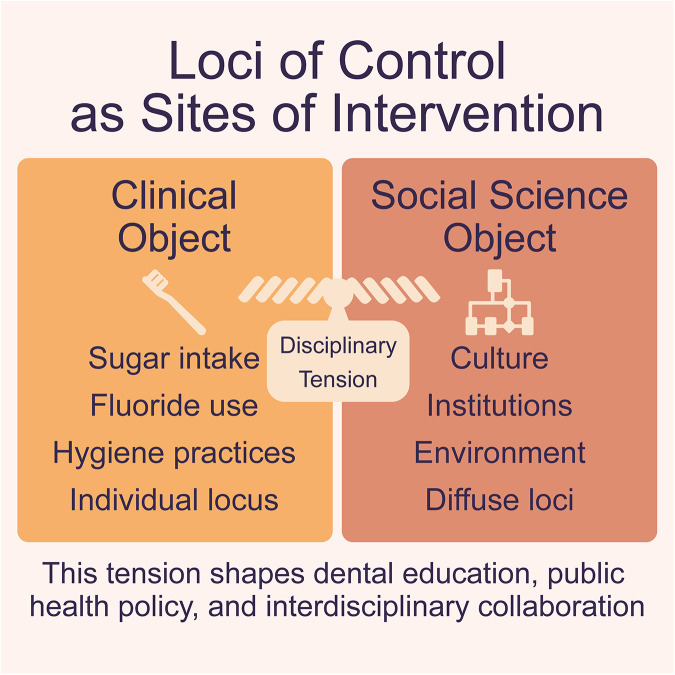
Loci of control as sites of intervention: contrast of individual and structural approaches.

There are tensions between clinical and social science orientations toward such a concept that have significant implications for dental education, public health policy, and interdisciplinary collaboration. Figure 3 visualizes the disciplinary tension between sites of intervention identified within oral health research—from individual behavior to structural determinants. By placing these orientations along a shared axis, the figure highlights how interdisciplinary synthesis depends on bridging micro-and macro-level analyses. To realize the promise of interdisciplinary collaboration, we must accommodate both the rigor of behavioral science and the depth of social inquiry. Bridge concepts, scalar frameworks, and social science methods are essential tools for understanding oral health as a lived, social, and ethical phenomenon that diversely trained researchers can meaningfully engage.

To advance the field, institutions ought to invest in interdisciplinary training and support the integration of social science into dental education. This includes modules on qualitative methods, cultural theory, and concepts like power and inequality. While dental education increasingly introduces students to ideas like SDoH, there is still a gap between exposure and integration. Take *dental home* as an example—a foundational concept in dentistry that emphasizes the importance of a consistent, trusted clinical relationship. Sustaining such a relationship also requires navigating broader social realities: the ability to pay, mobility, the fear of judgment or shame, cultural expectations, and institutional access. In this sense, dental care is never only technical—it is also deeply social. Dental schools ought to design curricula and facilitate field experiences that reflect this reality.

Research must prioritize interdisciplinary studies on SDoH, using mixed methods and community engagement to ensure relevance. In practice, clinicians should adopt patient-centered care models, apply behavioral interventions and advocate for policies that reduce disparities. By embedding social science principles across education, research, and clinical practice, dentistry can move toward equitable, holistic care that improves population oral health.

**Figure 4:**
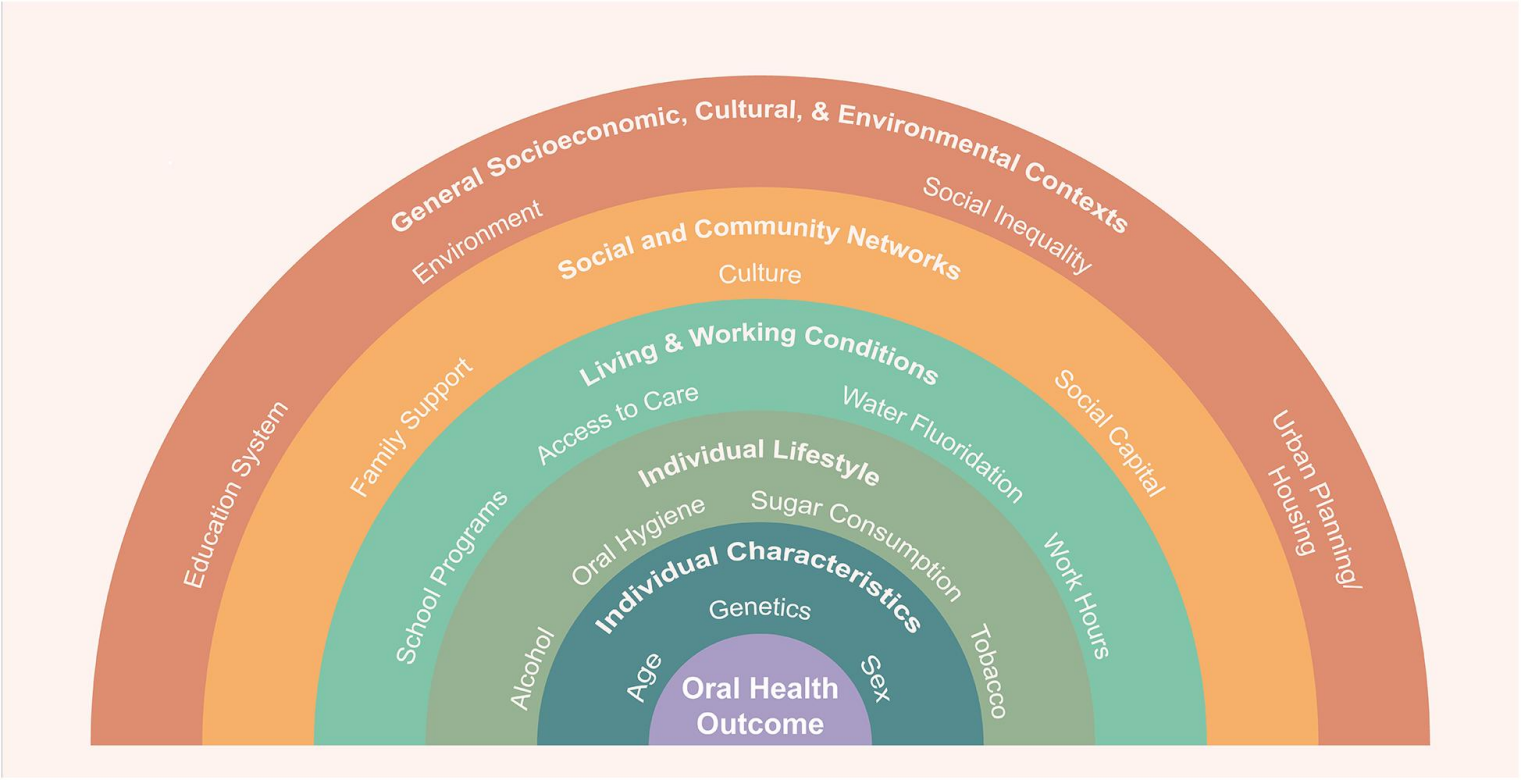
SDoH at scale; adapted from the Dahlgren and Whitehead rainbow model (116).

Social science becomes especially relevant when we shift focus from dentistry, the profession, to oral health as a societal goal. Figure 4 presents a framework for understanding oral health outcomes. The concentric rainbow bands illustrate how determinants of oral health operate at multiple, interacting levels—from individual characteristics and lifestyle behaviors to living and working conditions, community networks, and wider socioeconomic, cultural, and environmental contexts. This model highlights that oral health is shaped by biological and behavioral factors, social structures, cultural meanings, and policy environments that distribute resources and risk. While dentists and dental hygienists are on the front lines of patient care, their work is shaped by policies, economics, and social dynamics that extend beyond the clinic. The social sciences contribute essential insight into how these larger forces influence behavior, trust, access, and outcomes.

Clinical and translational research links biology to outcomes but cannot reduce disparities alone, as advanced treatments often widen equity gaps because of barriers to accessibility. Individual behaviors—diet, hygiene, smoking, literacy—require clinical and behavioral research to inform interventions. Living and working conditions such as housing, education, and healthcare access demand interdisciplinary study, while community networks and cultural norms highlight the need for culturally responsive, community-based approaches. These layers underscore that advancing oral health equity requires interventions across biological, behavioral, social, and structural domains.

## 5 Summary and future directions

This review has traced the evolution and diversification of social science contributions to oral health and dentistry, emphasizing recent articles published between 2015 and 2025. Across the sections, some key insights emerge. First, contemporary research has expanded engagement with qualitative methods and tends to emphasize key populations of interest, including immigrants, caregivers and children, and the elderly—populations whose oral health experiences are shaped by cultural logics, structural constraints, and institutional access. Second, qualitative and mixed methods approaches are central to this work, offering high-resolution accounts of lived experience and enabling researchers to interrogate the social dimensions of oral health with greater nuance. Building on earlier reviews, this paper provides a conceptual synthesis that maps existing contributions and outlines actionable strategies for integrating diverse disciplinary perspectives.

The scarcity of social science oral health research from low and middle-income regions reveals a structural gap in global research production. Addressing this disparity will require long-term collaboration and capacity-building initiatives that support locally grounded, interdisciplinary inquiry.

A central limitation in much qualitative oral health work is the loose or implicit use of theory. Future studies should clearly articulate theoretical frameworks drawn from the social sciences to sharpen analytic focus and enable comparison across studies. Valuable theoretical frameworks have been developed across multiple domains of social inquiry, e.g., ecological and social environment models; practice theory and intersectionality; embodiment and phenomenology (17, 113–115). Many of these frameworks have already proven fruitful in oral health research. Explicit theory-building of this kind will allow qualitative findings to inform intervention design and policy more directly, particularly in geriatric dentistry and community-based oral health promotion, where conceptual clarity can guide equitable and sustainable practice.

The field has also seen the emergence of new conceptual tools. Bridge concepts are useful in linking clinical phenomena to broader social theory. These concepts facilitate interdisciplinary collaboration by providing shared analytical vocabularies. They should be further elaborated upon and operationalized to guide both research and practice. Looking ahead, future frameworks should build on these insights by the co-production of research conventions that connect individual behavior to structural determinants in a way that is persuasive to diversely trained members of the oral health research community. Scalar approaches offer a promising model, emphasizing the need to consider influences at multiple levels—from the individual to the global, with a call for more middle-range efforts. Researchers should also continue to recruit pre-existing health frameworks that integrate existing knowledge systems into oral health practice.

In sum, the future of oral health research lies in its ability to engage with complex relationships—recognizing that more than a clinical outcome, oral health is a social, cultural, and ethical phenomenon. Social science perspectives do more than contextualize clinical findings; they can be emancipatory by exposing institutional blind spots, amplifying under-resourced voices, and reconfiguring care toward equity and dignity. Realizing this potential requires institutional changes—embedding qualitative and critical methods in curricula, funding community-partnered research, and valuing reflexive practices in clinical evaluation. When social inquiry is treated as central rather than peripheral, oral health research and practice gain the conceptual tools necessary for more impartially distributed, responsive, and theoretically grounded approaches to care.
